# Short Notes on Some of the Details of Sanitary Police

**Published:** 1855-12

**Authors:** Robert Druitt

**Affiliations:** Licentiate of the Royal College of Physicians, London.


### SHORT NOTES ON SOME OF THE DETAILS OF SANITARY POLICE.

By Robert Druitt, Licentiate of the Royal College of Physicians, London.

### No. II.—ON THE EFFICIENT REGISTRATION OF DISEASE.

In my preceding communication, I dwelt upon the expediency of a systematic record of disease by medical practitioners. This I did for two reasons: first, in order that the existence, the history and characters of all epidemic and contagious disease may be made known to public authority: this point I shall return to presently; secondly, in order that the various lesser maladies which arise from the presence of the great fever poisons may be traced to their source, and be thoroughly studied by the profession.

I am glad to find what I said on this latter point has excited some attention amongst my professional brethren; and as it is a matter of the utmost importance, I beg leave to repeat and enlarge upon some of the observations I made in the former number of these "Short Notes."

Fever poisons differ materially from poisons of a purely chemical nature, in this important respect—chemical poisons can operate on all persons, at all times. If we make due allowance for slight differences of constitution, we may say that arsenic and prussic acid are fatal to all men alike, and that the train of symptoms they produce may, in ninety-nine out of a hundred cases, be fairly predicted.

But fever poisons, instead of being purely *chemical*, and operating on all men alike, seem rather to be *zymotic;* that is, to act as ferments; and if so, of course it is necessary, in order for them to act, that there should be in the body some substance in the peculiar state which renders it fit to undergo fermentation. Yeast may be put into a barrel of beer, and will cause it to ferment so long as it contains free sugar; but when the sugar is exhausted, the yeast, *whatever other changes it may induce*, will not and cannot cause the alcoholic fermentation, nor any growth of fresh yeast.

So the poisons of small-pox and scarlet-fever, whatever other effects they may induce, can only produce their distinct and typical maladies respectively, when the constitution contains the unknown something upon which they act as ferment, and out of which they reproduce themselves.

One hundred persons shut up in a house, and equally subjected to the vapour of prussic acid or of arsenic, would probably all suffer pretty much alike. But one hundred persons exposed to the scarlet-fever poison would suffer very differently: some might have an attack fatal within twenty-four hours; some would escape absolutely uninjured; while many would exhibit the malady in an infinite number of degrees of intensity.

But I contend that, just as a soldier, who cannot fire his musket because his ammunition is spent, may yet use it as a very effective weapon in the form of a club, so the great fever poisons, although they may not be able to produce their full effects, because the body they act upon is deficient in the peculiar material necessary, yet may produce a horde of lesser and irregular effects. And I contend that the study of these is a matter of the deepest interest and importance. But this can only be carried out by records shewing where and when the great fever poisons prevail, and what other maladies prevail at or about the same times and places.

The importance of the subject, both to the profession and to the public, is immense; since, if my theory be correct, we may often be able to trace the origin of various perplexing maladies to their source, and, instead of purging the patient, may send for a builder to purify the drains of the house.

I gave in my last paper some cases in which patients, who were not ill enough to keep their bed, yet had a train of similar symptoms, explicable only on the hypothesis that they had imbibed a feeble dose of the poison of typhoid fever. Let us give some more instances of the same kind.

At Midsummer 1854, the three children of Mr. T., residing at

No. 21, —— Terrace, in a certain part of the suburbs, had scarlatina. I was consulted about one of them, who had dropsy afterwards. They all did well. The house was well situated and well drained; but it was the last house in the terrace; beyond it the ground was being prepared for building, and was (as usual) made the receptacle for all kinds of filth. Within ten yards of the house was a disgusting heap of offal, cinders, intestines of chickens, and decayed vegetables, permitted to be *shot* as rubbish, to raise the ground. This, by the bye, I believe to be a common practice, and a possible source of danger in new neighbourhoods, as I shall point out in a future number of these "Notes."

In November 1854, long after the children were well, the mother was one day seized with feverishness. This had entirely subsided on the next day, when I saw her; but she complained of sore-throat, and her uvula was sloughing. It did slough off, as completely as if it had been shaved off with scissors. But under the use of wine and bark she soon got well, *minus* her uvula.

In April 1855, a family residing at No. 19 in this terrace, were visited with a very imperfectly developed form of scarlatina. There was little or no rash, but sore throat and slight indisposition, followed by swelled glands and soreness of the nose. Four children suffered in this way; and although they were poorly for many days, yet not one was obliged to remain in bed more than one day.

Precisely at this time, I was sent for again to see Mrs. T., at No. 21. She had had another attack of feverishness and sore throat, and a round spot of the soft palate was sloughing. This made a round hole of the size of a fourpennny-piece, which is hardly closed to this day.

Now my impression is, that the attacks of sloughing in this lady's throat were caused by a mitigated dose of scarlatina poison, acting on a person not susceptible of the full effects of that poison. But this impression can only be ripened into certainty by the record of so many other cases of the same sort, as shall afford fair evidence of cause and effect.

On the 6th of July, 1855, I was sent for to see Lady B., at an hotel in —— Street, Piccadilly. I found her shivering, with violent headache and fever, a white tongue, and a red throat. On the following day all fever had vanished; there was no headache, the pulse was quiet, the skin cool, and there was desire for food and wine; but the throat was covered with a diphtheritic exudation, which began on the posterior third of the soft palate, and covered the tonsils and uvula. It formed quite a thick, tough, adhesive crust, the edge of which could be lifted up with the handle of a spoon, but it could not be detached without bleeding. The irritation and feeling of choking were intense; but there was no fever; and by the use of nourishment and common remedies, the patient was able to leave town on the 14th.

On the 13th, her maid had a precisely similar attack: sudden shivering, four-and-twenty hours of extreme fever, headache, and vomiting, followed by the most absolute apyrexia, and a throat covered with a tenacious exudation, which created immense irritation, but cleared off in five days.

At this time, scarlet fever was present in many houses at the end of the street adjoining Piccadilly; and there were two deaths from it, one on each side of the street.

In January 1855, scarlatina attacked two children in the family of a friend. In March, another child was attacked. At the same time with each of these attacks, the father of the children was seized with shivering, and slight but marked sore-throat; yet not enough to make him consider himself ill. This gentleman had never had scarlatina, though he had been often exposed to the infection of it.

In August and September 1854, during the cholera epidemic, there were several severe cases of diarrhœa amongst the family and servants of Mr. E., living near the Strand. In January 1855, diarrhœa amongst the children. In September 1855, one child had gastric fever severely. In October, the mother had diarrhœa. I was sent for, and learning the previous illnesses, inquired as to the drainage of the house, and existence or not of effluvia. I was told that the drains had been lately thoroughly repaired, but that there was some smell at times. This was examined into, and a closet-pipe, in the centre of the house, was found to be imperfectly trapped. This was attended to; but all November the mother suffered from diarrhœa at intervals; and at this time the father had febricula, from which he has not yet perfectly recovered.

There are some poisons, of which the lesser effects, such as occur when patients are not susceptible of the full effects, are known accurately enough. Such is the vaccine poison, from which at least three classes of effects may be produced: on the unprotected, the full vaccine vesicle running a twenty-one days' course; on the protected, either a spurious vesicle, which also runs a twenty-one days' course, or a mere boil, whose effects are over in from five to ten days. In every case of efficient vaccination, there is evidence that the patient's arm has been punctured with something besides a clean lancet; yet it is only in a certain class of persons that the typical and distinctive effects of the venom are shewn.

But in like manner, we want a knowledge of the lesser forms of the other great blood-poisons. I feel convinced that many anomalous maladies may be traced to these, and that our patients would be inconceivably benefitted, if we had the means of following up the investigation. For my own part, when diarrhœa, bilious attacks, or anomalous febriculæ prevail in a house, I always inquire whether the house can be at fault; and in many cases, I am sure that the best chalk mixture for diarrhœa is that which is mixed by the bricklayer's labourer*.

---

* Let me, by way of parenthesis, give this maxim. Whenever drains are cleansed, *see* that the filth is carted off the premises: else much of it will be mixed with earth, and laid down outside the new drain.

This knowledge, I contend, can only be attained in perfection by something like a *registry of disease,* founded on the daily observations of medical practitioners universally.

Leaving now the more strictly intra-professional view of the matter, let me say a few words on the registration of disease generally.

All legislation ought to be based on facts. The verification and collection of facts is therefore one of the primary duties of a wise legislature. No object is so worthy of legislation as the preservation of health and life. The accumulation of facts, regarding health and life, ought to go before and guide legislation thereon.

I might go on, and fill a page with an alphabet of truisms, or of what ought to be truisms, but have scarcely yet arrived at that dignity.

But, respecting disease, we are content at present to take our facts from records of mortality only. In a more advanced era, we may hope for a register of disease that is not mortal. I have shewn in my last, by what very simple machinery the elementary facts might be recorded. I am prepared with an equally simple plan by which they may be collected, for professional and legislative use; at all events, there is nothing extravagant in saying, that the officers of health under the new Metropolitan Local Management Act ought to have cognisance, not merely of the mortality, but of the diseases prevalent in their respective districts; and for this purpose, something like the plan I have proposed is indispensable.

The legislature has already interfered in the most summary way with regard to one disease, *small pox;* but even with regard to this, two things are wanting to render legislation effective. One is a registration of the times and places at which the disease prevails, whether mortal or not; the other, some provision for carrying the vaccination act into working effect, and for taking care that the penal clauses are not laughed at, as they now are, both by the profession and the public.

But in the natural order of things, it is quite right that scarlet fever should be next taken notice of. It is a malady whose intrinsic mischiefs are aggravated by the almost slavish and unreasonable fears it excites, especially amongst the upper classes of society: and the present mortality from it exceeds that of small pox. In four years (1849-52) there were 23,627 deaths from small-pox, 56,672 from cholera, and 58,014 from scarlatina in England. In the parish of St. George, Hanover-square, from 30 to 40 deaths from scarlatina occur annually. What an amount of disease, not fatal, this implies! Scarlatina is a malady most insidious; the after consequences are most disastrous, and, as I contend, it is capable of producing many lesser and ill-recognized effects on wide classes of persons, who yet could not be said to suffer from developed scarlet fever. As the basis of preventive measures, a full estimate of the extent of the disease is indispensable; and this demands the adoption of some such measure as I advocate for the registration of disease.

Curzon Street, Mayfair, December 1855.

